# Characterizing Counterion-Dependent Aggregation of Rhodamine B by Classical Molecular Dynamics Simulations

**DOI:** 10.3390/molecules28124742

**Published:** 2023-06-13

**Authors:** Giacomo Fanciullo, Silvia Orlandi, Andrey S. Klymchenko, Luca Muccioli, Ivan Rivalta

**Affiliations:** 1 Dipartimento di Chimica Industriale “Toso Montanari”, Alma Mater Studiorum, Università di Bologna, Viale del Risorgimento 4, 40136 Bologna, Italy; 2 Laboratoire de Bioimagerie et Pathologies, UMR 7021 CNRS, Université de Strasbourg, 74, Route du Rhin, 67401 Illkirch, France; 3 ENSL, CNRS, Laboratoire de Chimie UMR 5182, 46 Allée d’Italie, 69364 Lyon, France

**Keywords:** Rhodamine B, dye aggregation, fluorinated tetraphenylborate, molecular dynamics, force field parametrization

## Abstract

The aggregation in a solution of charged dyes such as Rhodamine B (RB) is significantly affected by the type of counterion, which can determine the self-assembled structure that in turn modulates the optical properties. RB aggregation can be boosted by hydrophobic and bulky fluorinated tetraphenylborate counterions, such as F5TPB, with the formation of nanoparticles whose fluorescence quantum yield (FQY) is affected by the degree of fluorination. Here, we developed a classical force field (FF) based on the standard generalized Amber parameters that allows modeling the self-assembling process of RB/F5TPB systems in water, consistent with experimental evidence. Namely, the classical MD simulations employing the re-parametrized FF reproduce the formation of nanoparticles in the RB/F5TPB system, while in the presence of iodide counterions, only RB dimeric species can be formed. Within the large, self-assembled RB/F5TPB aggregates, the occurrence of an H-type RB-RB dimer can be observed, a species that is expected to quench RB fluorescence, in agreement with the experimental data of FQY. The outcome provides atomistic details on the role of the bulky F5TPB counterion as a spacer, with the developed classical FF representing a step towards reliable modeling of dye aggregation in RB-based materials.

## 1. Introduction

**Figure 1:**
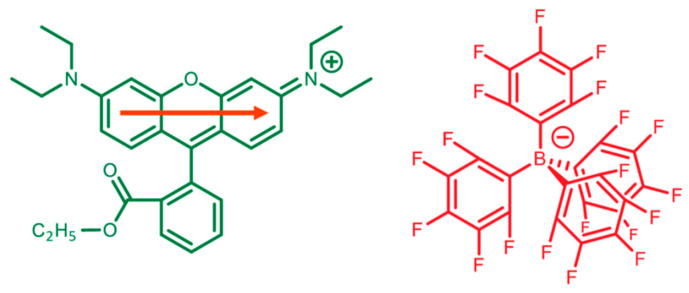
Target molecules of this work, including Rhodamine B ethyl ester (RB, **left**), with its transition dipole moment parallel to the xanthene plane, and tetrakis (pentafluorophenyl) borate counterion (F5TPB, **right**).

Rhodamine B (RB) is a xanthene derivative (see Figure 1, depicting RB ethyl ester) with remarkable optical properties, the most important being its high fluorescence quantum yield, FQY [1], and is, thus, exploited in a wide range of technological applications such as fluorescent probes [2], chromic materials [3], thermal lensing [4], optical thermometers for biological systems [5], and artificial light-harvesting (LH) nanomaterials. Regarding the latter, RB-alkylated derivatives have been successfully employed as the fundamental unit of photoactive dye networks encapsulated in polymeric nanoparticles, showing fascinating collective optical properties such as a photo-induced reversible on/off switching of single-particle fluorescence [6]. This outcome suggested that the electronic excitations can easily migrate throughout a network of RB dyes via energy transfer (ET) and, therefore, enable a single quenching molecule (if present) to suppress the fluorescence of the whole nanoparticle. Indeed, these RB-based polymeric nanosystems have been in turn exploited to create giant LH nano-antennas [7], in which solar light can be harvested and transferred from RBs to a single acceptor molecule that gains an impressive enhancement (up to 10^4^) of emission intensity.

The synthesis of LH nanoparticles exploits the hydrophobic character of RB derivatives, which allows for easy encapsulation within polymeric media but also provides RB with the ability to strongly interact with bulky and hydrophobic counterions [6,7]. These counterions can be exploited as spacers between dyes with the purpose of reducing RB-dimer formations, responsible for fluorescence quenching even in a solution [8]. Interestingly, the counterion has turned out to be a powerful modulator of RB network formation and consequently of nanosystem photoactivity [9]. Indeed, the counterion size, which clearly affects the dimerization extent, in conjunction with its hydrophobicity and the polymer, can be used to fine-tune the optical properties by modulation of dye encapsulation and dispersion in the polymer matrix [10].

A counterion that has proved successful in driving the formation of efficient RB-based LH networks is F5TPB, a derivative of tetraphenylborate bearing perfluorinated phenyl groups, whose structure is depicted in Figure 1. F5TPB promotes the formation of networks in which RB units are very close to each other (thus allowing for efficient ET among dyes) but, at the same time, prevents an RB dimerization that completely quenches fluorescence. This capability of F5TPB has been observed not only in polymeric nanoparticles [6,7,9] but also in a solution (i.e., in the absence of a polymer). In particular, when added to aqueous solutions of RB ethyl ester, F5TPB provokes the formation of colloidal nanoparticles constituted by the clustering of RB^+^ and F5TPB^–^ ions showing the same efficient ET observed in polymeric nanosystems [11]. Moreover, if compared with RB water solutions in the presence of iodide counterions, RB/F5TPB aggregates have only slightly decreased FQY (diminishing from 30% to 20%), which can be, however, increased to ca. 60% by augmenting the fluorinated counterion size [11]. These experimental data indicate that F5TPB effectively acts as a spacer between RB monomers such that ET processes are still effective and fluorescence is not fully suppressed. 

Achieving insights into the chromophore arrangements within the dye networks is very challenging from an experimental point of view. Indeed, while the presence of particle blinking could support the picture of an arrangement in which the dyes are quite close to each other and an estimate of the mean dye-dye distance could be inferred from spectroscopic measurements, such as fluorescence anisotropy decay [6], atomistic details of the network structure are completely out of reach. 

Since RB aggregation is a phenomenon that controls the optical properties of RB-based materials, it is crucial to understand and control this assembly process to achieve effective technological applications of RB dyes. In order to achieve fundamental insights into the atomistic structure of RB/counterion aggregates, classical molecular dynamics (MD) simulations certainly represent a powerful tool. However, appropriate FFs need to be developed, possibly validating them with experimental evidence. While MD simulations have been used to simulate the RB absorption on clay minerals [12], graphene [13], and membranes [14] and to reproduce the diffusion in polyelectrolyte solutions [15], to the best of our knowledge they have not been used to describe the interaction of RB with bulky hydrophobic counterions in a solution. Thus, the characterization of the key molecular interactions responsible for the formation of dye networks suitable for LH purposes remains unexplored.

In this paper, we present and validate a force field (FF) for the modeling of self-assembly in RB/F5TPB systems in a water solution based on the widely used generalized AMBER force field [16] (GAFF). A full parametrization of the RB unit and an ad hoc refinement of the RB Lennard-Jones (LJ) parameters were performed, aiming at a reliable reproduction of the RB-RB interactions, using reference density functional theory (DFT) calculations. The reliability of our FF is judged by its ability to reproduce experimental studies on the influence of counterion type and alkyl RB substituents. 

## 2. Computational Methods

In this work, all quantum mechanical (QM) calculations have been performed at the DFT level using Gaussian16 software [17] with the B3LYP functional [18] by using D3 Grimme’s energy corrections to model dispersive interactions [19] and the 6-311+G* basis set. This function has been previously used for characterizing the photophysical properties of different rhodamines, including RB [20]. All molecular mechanics (MM) calculations have been performed using NAMD 2.12 software [21].

### 2.1. Force Field Parameters

**Figure 2:**
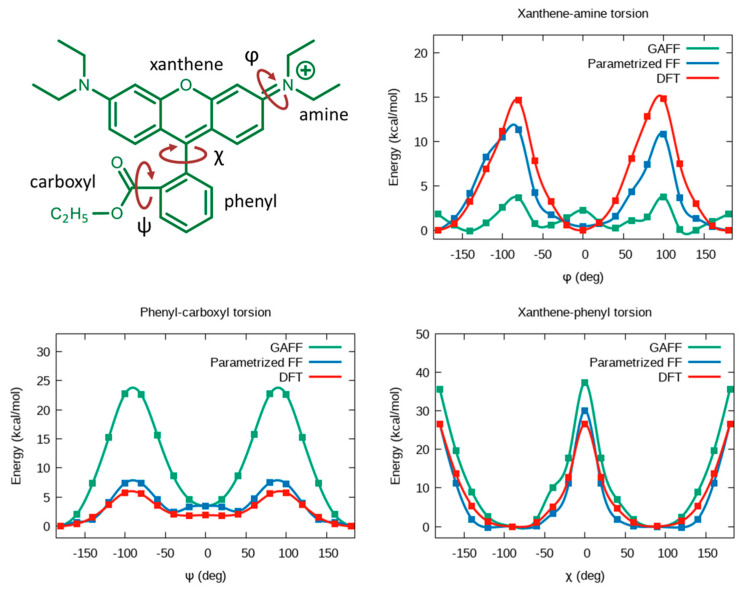
RB dihedral angles selected for parametrization and corresponding potential energy profiles calculated with the original and modified GAFF force fields, compared with DFT (B3LYP-D3//6-311+G*) calculations used as reference. The MM energy of each point is calculated at the DFT-optimized geometry.

The FF parameters for RB ethyl ester (see Figure 1, for conciseness, referred to as RB hereafter) have been obtained utilizing the following procedure. First, DFT calculations were used to optimize the geometry and to calculate the corresponding ESP charges. In order to evaluate if the standard GAFF parameters provided a reliable minimum energy ground state (GS) structure of RB in a vacuum, we compared the DFT-optimized geometry with the MM one (involving GAFF standard parameters and ESP charges). Since at the MM level, several bonds of the xanthene moiety were found to deviate by more than 0.01 Å (up to 0.04 Å) from DFT, we defined new atom types to describe all xanthene atoms (and others, see Supporting Information, Supplementary Materials, and Figure S1) and replaced the equilibrium bond distances in the force field with the DFT ones, leaving unaltered the force constants. Then, we adjusted the MM parameters of the xanthene-phenyl, xanthene-amine, and phenyl-carboxyl torsional potentials, as reported in Figure 2. It is worth mentioning that for the xanthene-amine torsion, we considered only one angle for the parametrization since the two amine groups present in RB are chemically equivalent (symmetric with respect to the symmetry plane containing the phenyl group and dividing the xanthene plane). For each dihedral, the total energy profile for the torsions has been calculated at the DFT level by constrained scans, i.e., evaluating the GS total energies every 10° of the selected dihedral angles and allowing relaxation of all the remaining internal coordinates. The corresponding free energy profiles at the molecular mechanics level were obtained with the adaptive biasing force method [22], using different dihedrals as collective variables in NVT simulations of a single RB conducted at 300 K. The force field parameters were iteratively modified until the DFT scan and the MM free energy profiles coincided within an error of 0.2 kcal/mol in the region of the minima (see ref. [23] for further details).

Regarding the MM parameters of the counterions, the standard GAFF parameters were used for iodide ions while for the F5TPB molecule, a minimal refinement was performed. In particular, we computed the F5TPB minimum energy structure with DFT in a vacuum and the corresponding equilibrium distance and force constant for B-C stretching were incorporated in the FF, while for bending and torsions angles involving boron, we took the parameters for tetra-phenyl methane from the literature [24]. The vdW parameters for boron atoms were also taken from the reference [24]. The final set of parameters obtained for RB and F5TPB is available in the Supplementary Materials (see Tables S1–S6). For water molecules, the flexible variant of the simple point-charge model (spc/fw) [25] was employed since it well reproduces the dynamical and dielectric properties of bulk water.

### 2.2. Molecular Dynamics Simulations

Several independent MD simulations of RB molecules in water were performed in the presence of either F5TPB (MD 1–4) or I^−^ (MD 1–3) as a counterion. The initial simulation boxes, created using the Packmol package [26], contained 10 RB dyes, 10 counterions (F5TPB or iodide), and about 20,800 water molecules within a cubic box with a side length of 85 Å. The box dimensions were chosen such that all RB-RB distances in the initial configurations largely overcome the value of 6 Å, i.e., much larger than in an RB dimer. It is worth noting that the experimental RB concentrations used in ref. [11] are much lower than those considered in our MD simulations. This is due to the fact that MD simulations will be computationally unfeasible at the experimental concentrations, as they would require very high numbers of solvent molecules. However, MD simulations at high concentrations still represent a fundamental test for FF reliability, as discussed in Section 3.

For each simulation, a timestep of 1 fs was used, and the cutoff for LJ and direct space electrostatic was set to 12 Å. The particle mesh Ewald method for the electrostatics was used by setting a grid spacing of 1.5 Å. We carried out 1 ns of thermal equilibration (in the NVT ensemble at 300 K) followed by 2 ns of pressure equilibration (in the NPT ensemble at 1 atm) using the Berendsen barostat as implemented in NAMD. After the initial equilibration steps, the MD simulations were performed in the NVT ensemble, and the RB aggregation was monitored for 20 to 70 ns.

**Figure 3:**
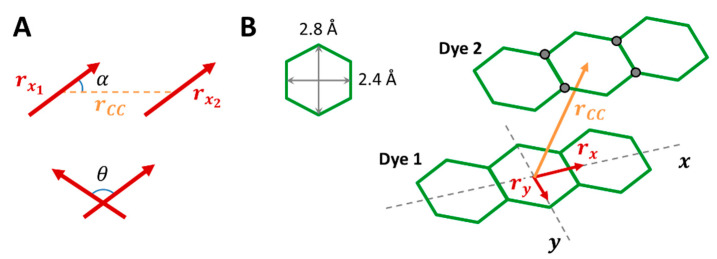
(**A**) Angles among the transition dipole moments of RB molecules (parallel to the x-axis, i.e., r_x_) employed for the dimer classification of H (0° < θ < 12°; 54.7° < α < 90°), J (0° < θ < 12°; α < 54.7°) and crossed-J (θ > 12°, any α) dimers. (**B**) Parameters employed for determining the π-stacking: the cutoff values for the center-center vector ($r_{CC}$) components were chosen to be $r_{x}$ < 4 Å and $r_{y}$ < 2 Å according to the typical dimensions of the hexagonal units of the xanthene plane (i.e., 2.4 and 2.8 Å), while the cutoff for the distances of the 4 atoms of the xanthene plane (labeled with grey circles) from the other xanthene plane was fixed to 4.5 Å, being the xanthene-xanthene distance predicted by DFT calculation of about 3.6 Å.

**Figure 4:**
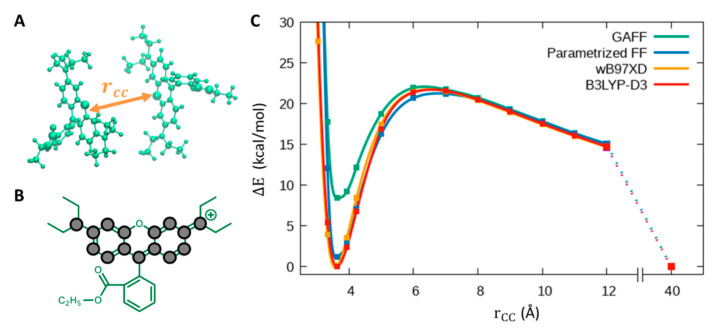
Parametrization of the RB-RB interactions in the π-stacked H dimer. (**A**) Representation of two RB molecules approaching to form an H dimer. (**B**) RB chemical structure, with grey circles corresponding to atoms for which the LJ parameters have been modified. (**C**) Intermolecular energy of the RB-RB system reported as a function of the xanthene-xanthene inter-plane distance (i.e., the $r_{CC}$ vector, orange arrow in panel **A**), with the energy of the two RB molecules at a distance of 40 Å set as reference, i.e., $∆E=E\left(r_{CC}\right)-E(40)$.

To quantify the time evolution of the RB dimerization in our simulations, we defined an “interacting” RB dimer as each RBs couple in which the center-center distance between the xanthene planes ($r_{CC}$, see Figure 3) falls below the cutoff value of 6 Å, which represents a distance at which the RB-RB interaction is already of attractive type in the stacked dimer in a vacuum (see Figure 4). This cutoff distance also ensures the exclusion of dimer configurations that could involve the presence of an F5TPB counterion between the two RBs. Moreover, considering that the RB transition dipole moment lies on the xanthene plane and parallel to its long axis, dimers have been classified as H, J, and crossed-J dimers, depending on the reciprocal orientation of the xanthene axes of each RB, see Figure 3A: we considered parallel dimers as having 0° < θ < 12° and crossed-J dimers as having θ > 12°, while for parallel dimers, we extracted the angle α to classify them as H (54.7° < α < 90°) or J (α < 54.7°). 

Another relevant feature to monitor is the formation of any type of π-stacked dimers because they are expected to act as fluorescence quenchers. [27] To quantify their presence we considered a combination of geometrical parameters including two components of the center-center vector and the interplane distance of the xanthene fragment depicted in Figure 3B: we considered a “stacked dimer” to be each RB pair having (i) the components of the $r_{CC}$ vector along the x and y direction within 5 and 2 Å, respectively, and (ii) distances below 4.5 Å separating the 4 black labeled atoms of one dye from the xanthene plane of one other.

In order to evaluate the formation of RB-RB clusters within nanoparticles eventually involving counterions, the sum of dye-dye distances, defined as the module of $r_{CC}$ vectors, was determined and monitored along the MD trajectory.

## 3. Results and Discussion

### 3.1. Force Field Development

In this work, we studied RB assembling in water solutions containing RB and either F5TPB or iodide counterions, with particular attention to dimer formation. To this aim, the first necessary step is to accurately parametrize the geometrical flexibility of the RB molecule. On the other hand, the high symmetry of the F5TPB molecular structure combined with its almost spherical shape makes its bulkiness largely independent from the intramolecular motions, so a fine parametrization for the counterion is not required. 

Thus, we first developed FF parameters for RB that would reproduce its conformational energies as computed at the DFT level. Figure 3 shows the comparison between such DFT torsional energy profiles (involving the dihedral angles described in Section 2 and the corresponding ones calculated at the DFT geometries at the MM level, either using the original GAFF or the re-parametrized FF. Clearly, the reparameterization was crucial for the correct description of the phenyl-carboxyl torsions since GAFF predicted quite large torsional barriers. Notably, the opposite trend is observed for the xanthene-amine rotation. This failure of GAFF probably arises from the fact that GAFF parametrization was performed on a database of neutral molecules, and it is likely that the standard torsional parameters are inadequate for describing the torsions in charged aromatic systems such as RB.

Since we were also interested in evaluating the dimerization of two positively charged RB molecules, we focused on the parametrization of the van der Waals interactions for two approaching RB molecules. We restricted the analysis to the Lennard-Jones (LJ) parameters of the xanthene moiety in order to correctly describe the binding energy of two approaching RB molecules, taking as a reference case the formation of the stacked configuration with the largest π-stacking, i.e., the H-aggregate dimer. We chose to take as reference the interaction energy given by the DFT calculations using Grimme’s dispersions [19], and we compared the B3LYP-D3 results with those obtained from the ωB97XD functional [28], which already includes dispersive interactions. First, we tested the performance of GAFF LJ parameters (and DFT ESP charges) on the potential energy surfaces (PES) related to two RB molecules approaching to form a stacked H dimer. To calculate the PES, we started from the dimer geometry optimized in vacuum at the B3LYP-D3 level and we varied the RB-RB distance leaving unchanged both monomer geometries. At each distance, we computed the DFT and MM total energies (all calculations were performed in a vacuum). The PES obtained in such a way is reported in Figure 4: being RBs positively charged, one clearly sees that to form a dimer, stabilized by the van der Waals interactions, the two dyes first must overcome a potential energy barrier arising from the Coulomb repulsive interaction. While the B3LYP-D3 and ωB97XD functionals produce almost identical PESs, it is immediately evident that GAFF, although reproducing the DFT profiles qualitatively, significantly underestimates the depth of the potential well with respect to DFT. To correct for such discrepancy, we uniformly increased the LJ parameters ε describing the strength of vdW interactions only among the atoms belonging to the xanthene moiety (see Figure 4B) until the QM PES was correctly reproduced: a good agreement was found upon doubling those parameters. 

### 3.2. Simulating the Aggregation of Rhodamine B Ethyl Ester in Water

**Figure 5:**
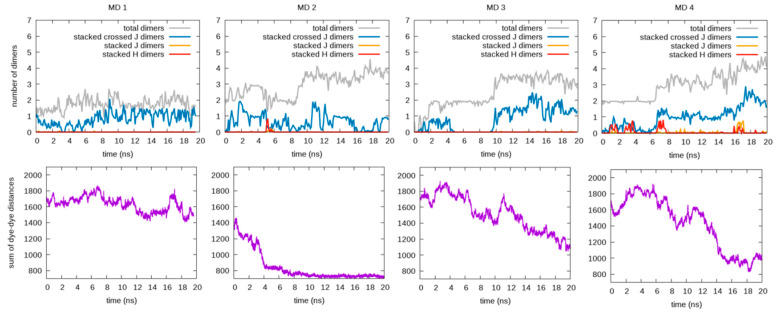
Analysis of four independent MD simulations carried out in the presence of the F5TPB counterion. The plots in the first row show the time evolution of the total number of dimers (gray lines) and, among them, the number of π-stacked dimers, being H (red lines), J (yellow lines), and crossed-J (blue lines) types. The plots in the second row show the time evolution of the sum of the $r_{CC}$ distances, which decreases when large aggregates are formed. The time-evolving data are reported as the mean values within time intervals of 0.1 ns.

**Figure 6:**
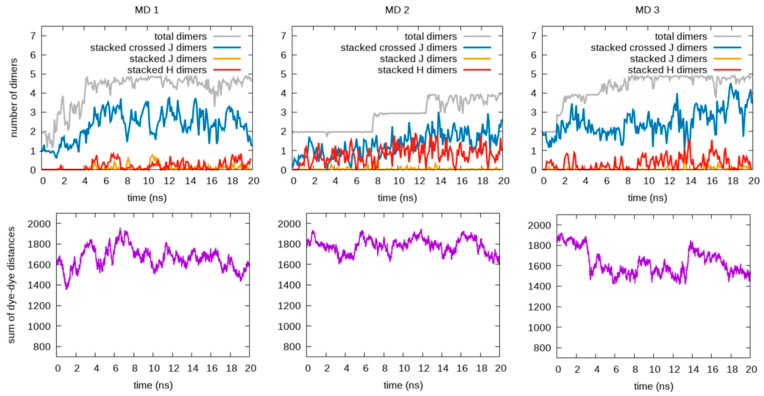
Analysis of three independent MD simulations carried out in the presence of the iodide counterion. The plots in the first row show the time evolution of the total number of interacting dimers (gray lines) and, among them, the number of π-stacked dimers, being H (red lines), J (yellow lines), and crossed-J (blue lines) types. The plots in the second row show the time evolution of the sum of the $r_{CC}$ distances, which decreases when large aggregates are formed. The time-evolving data are reported as the mean values within time intervals of 0.1 ns.

**Figure 7:**
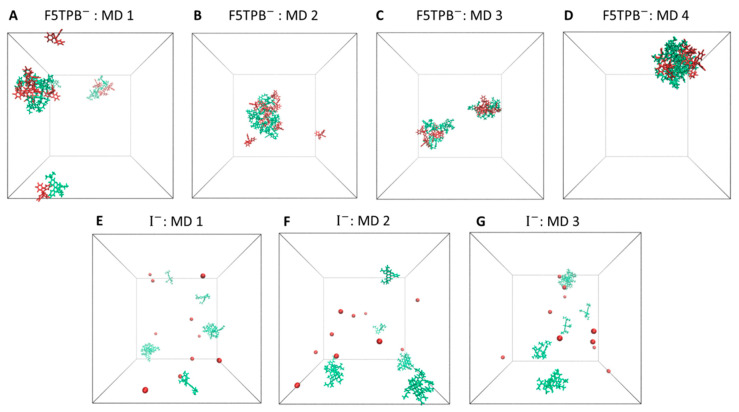
Representative snapshots were extracted at the end (i.e., at 20 ns) of the three independent MD simulations of the RB/F5TPB (panels **A**–**D**) and RB/I (panels **E**–**G**) systems. RB molecules are depicted in green, counterions in red, and solvent molecules are omitted for clarity.

Once the new force field parameters were obtained, we tested their predictions for RB aggregation in ionic water solutions. Figure 5 and Figure 6 show the results of the independent MD simulations carried out (using the re-parametrized FF of RB monomers and RB-RB interactions) for each of the counterions considered, F5TPB and iodide. In particular, the number of dimers (interacting RB pairs, including H, J, and crossed-J π-stacked conformations) formed along the MD trajectories (extracting one frame every 1 ps) are reported as moving averages (on time windows of 0.1 ns). The time evolutions of the sum of the whole set of $r_{CC}$ distances are also reported in Figure 5 and Figure 6, being a useful parameter for qualitative evaluation of the aggregations involving a large number of RB, i.e., this sum decreases when large aggregates are formed. Representative snapshots reporting the typical structure of RB aggregates in these MD simulations are depicted, instead, in Figure 7.

By comparing the trends in Figure 5 for F5TPB with those in Figure 6 for iodide, it clearly appears that only the F5TPB counterion can drive the formation of large aggregates (see representative configurations in Figure 7A–D). Interestingly, in our independent MD simulations with F5TPB counterion, we observed the formation of either a single RB/F5TPB aggregate comprising all RBs (namely a “full aggregate”, see MD 2,4) or two separated aggregates (MD 1,3), see Figure 7B,D and Figure 7A,C, respectively, allowing structural characterization (with atomistic resolution) of different dimeric configurations within these aggregates, as discussed below. While the formation of a full aggregate is not observed in all 20 ns simulations, the prolongation of MD 1,3, reported in Figure S2 in the Supplementary Materials, showed this can occur in these cases within 70 ns. This extensive aggregation (see also the lowering of the sum of dye-dye distances in Figure 5 and Figure S2) is in line with the spontaneous formation of nanoparticles in water experimentally observed in ref. [11]. Thus, these simulations provide important insights at the atomistic level and interesting trends as a function of the counterion involved. Indeed, when the iodide counterion is considered, dimer formation is still observed but the formation of large RB or RB/iodide aggregates is not. Accordingly, the lower panels in Figure 6 show that the sum of dye-dye distances is constantly large along all MD simulations. These results are in line with the absence of RB nanoparticle formation in experiments [11]. Notably, such RB aggregates are not formed in the presence of iodide despite the large RB concentration employed in our MD simulations.

Other than inducing the spontaneous aggregation of RB, we clearly observed the ability of the F5TPB counterion to act as a spacer between RB monomers within the aggregate, preventing the H dimerization responsible for fluorescence quenching. Indeed, another striking result is that, while in the presence of the iodide counterion, the formation of π-stacked dimers is present (three to four stacked dimers are always observed, including one of the H-type, see Figure 6), in the presence of F5TPB. Despite the occurrence of large RB/F5TPB aggregates, the formation of π-stacked RB dimers is quite limited and the appearance of H-type dimers is a rather rare event. In particular, while up to four RB interacting dimers are formed within large RB/F5TPB aggregates (see Figure 5), only one (and rarely two) among them can be really considered as π-stacked, generally being the crossed-J type. In fact, a single stacked H dimer stable enough to persist for about 1 ns (then transiently converted into a J and finally into a crossed-J dimer), has been observed only in MD 2,4 (see Figure 5). This result, i.e., the absence of stable H aggregates in the presence of F5TPB, is strengthened by recalling that our parameterization for the RB-RB LJ interaction is based on the correct description for the formation of a stacked H dimer.

Therefore, our simulations strongly suggest that within RB/F5TPB aggregates there is only a small amount of π-stacking dimerization, corroborating the role of an F5TPB counterion as an RB-RB spacer able to prevent the complete quenching of RB fluorescence. In fact, the experimental FQY for RB/F5TPB aggregates in water is ca. 20%, only slightly smaller (namely ca. 30%) than in the presence of iodide [11]). Moreover, among π-stacked conformations, there is a preference for crossed-J dimers and a dislike for H and J dimers, in full agreement with experimental evidence that F5TPB counterions allow an efficient energy transfer between RB dyes while preventing complete fluorescence quenching.

**Figure 8:**
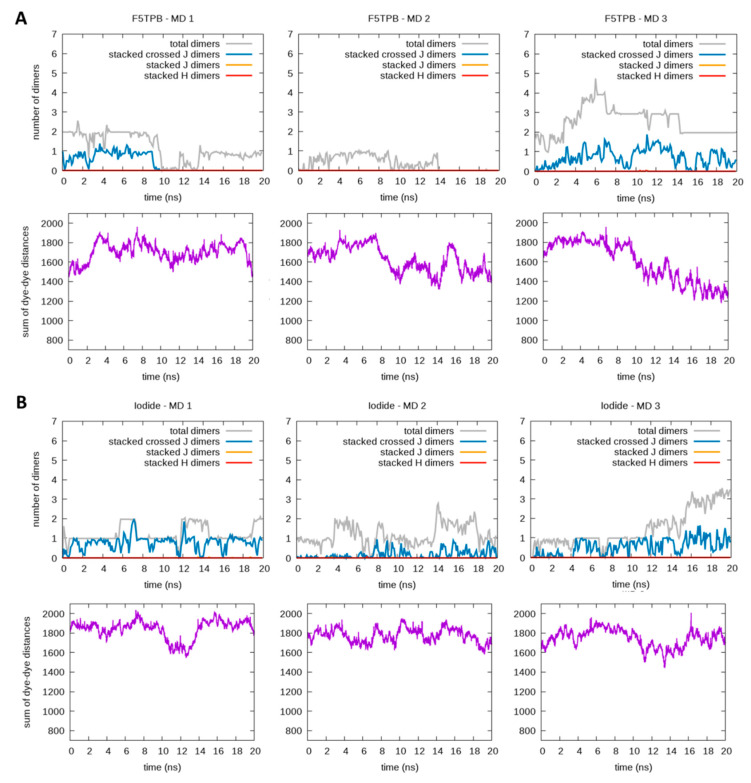
Analysis of three independent MD simulations carried out in the presence of either F5TPB (panel **A**) or iodide (panel **B**) counterions with standard GAFF. For each panel, the plots in the first row show the time evolution of the total number of dimers (gray lines) and, among them, the number of π-stacked dimers, being H (red lines), J (yellow lines) and crossed-J (blue lines) types. Instead, the plots in the second row show the time evolution of the sum of dye-dye distances, which decreases when large aggregates are formed. The time-evolving data are reported as the mean values within time intervals of 0.1 ns.

In order to evaluate the effect of our parametrization with respect to standard GAFF, we also performed six simulations with GAFF, three for each (F5TPB and iodide) counterion, as shown in Figure 8. We found that large aggregation in the presence of the F5TPB counterion is also partially predicted by GAFF parametrization, with the formation of two large aggregates being observed, while a full aggregate comprising all RBs is never formed in 20 ns. The absence of large RB clustering in the presence of iodide observed with the re-parametrized FF is very similarly found with the GAFF parametrization while the dimerization extent predicted by GAFF is reduced (only one π-stacked dimer is observed on average) and no H dimers are formed. This is consistent with the fact that, as discussed in Section 3.1, the standard GAFF underestimates the π-stacked H dimer binding energy. The effect of underestimating vdW interactions is also visible within the RB/F5TPB aggregates, in which much less dimerization is observed with GAFF and very few π-stacked dimers are formed if compared with the re-parametrized FF. 

In order to better understand how the increased attractiveness of vdW interactions given by our LJ re-parametrization affects the RB and F5TPB distribution within the aggregates, we compared the radial pair distribution functions related to both the RB-RB and RB-F5TPB pairs obtained from the MDs performed with our new FF and with GAFF, see Figures S3 and S4 in the Supplementary Materials. The mean pair distribution functions were calculated using VMD software [29] as averages performed on the last nanosecond of the MD trajectories, i.e., when the RB/F5TPB aggregates were completely formed. The enhanced formation of π-stacked crossed-J dimers given by the re-parametrized FF is clearly reflected by the modifications appearing in the RB-RB distribution functions. Indeed, while in the two GAFF simulations (with π-stacking observed only in one of the two simulations for a crossed-J dimer surviving for 10 ns) the distance corresponding to the highest probability of finding another RB unit is observed at about 7.5 Å (see Figure S3B in the Supplementary Materials), in the three simulations performed with the re-parametrized FF, the distance is reduced to 5 Å in MD 1 and to about 4 Å in MD 2–3 (see Figure S3A in the Supplementary Materials). Correspondingly, the mixed RB-F5TPB distribution functions show that the probability of finding an ion-pair at high distances is larger with the re-parametrized FF (see Figure S4A,B in the Supplementary Materials), consistently with the observation of larger aggregates.

Overall, the occurrence of RB/counterion full aggregates only in the presence of F5TPB and the rare (and transient) appearance of H dimers (despite the significant number of stacked J dimers) within large RB/F5TPB aggregates are robust predictions of our re-parametrized FF and represent important progress towards the characterization of an atomistic resolution of RB aggregation processes in a solution.

## 4. Conclusions

In this work, we developed a classical FF able to describe the aggregation of cationic RB dyes in water induced by the presence of hydrophobic and bulky counterions, such as F5TPB. In particular, a new FF, based on the standard GAFF parametrization, has been developed for an ethyl ester derivative of Rhodamine B and validated by comparing simulations of water-solvated RB aggregates in the presence of either iodide or F5TPB hydrophobic counterions, which feature different experimental behaviors.

In particular, parameters related to the RB conformational space and the RB-RB van der Waals interactions have been involved in the re-parametrization. In both cases, DFT-computed potential energy surfaces were taken as a reference. Indeed, we found that standard torsional parameters needed to be re-parametrized because they could not properly describe some torsions in a charged aromatic system such as RB dye. Moreover, GAFF parameters of RB reproduce only qualitatively the DFT-computed PES for the formation of an RB dimer with π-stacked xanthene moieties (i.e., the H-type aggregate responsible for the reduction of FQY); therefore, we performed a refinement of the force field LJ parameters to reproduce it quantitatively.

In order to validate the re-parametrized FF, we relied on the experimental evidence that while in the presence of iodide counterions, the RB dyes are soluble in water and the RB/F5TPB ion pairs undergo assembling that leads to nanoparticle formation, with a corresponding decrease of the FQY of the sample. Accordingly, our MD simulations predicted the formation of whole aggregates exclusively for the bulky F5TPB counterion, while in the presence of iodide, only RB dimeric species can be obtained. The same trend can be found adopting the standard GAFF but with a relevant difference since GAFF simulations showed less tendency to form aggregates. Therefore, only by employing the re-parametrized FF can a full RB/F5TPB aggregate be observed. This represents an important outcome since only within such full aggregates (i.e., only after the FF re-parametrization), could we detect the transient formation of an H-type dimer, which is the species that explains the decrease of FQY observed experimentally. It is worth mentioning that, despite the re-parametrization of the RB-RB interactions being based on the PES for the formation of a π-stacked H dimer, the H dimerization was found to be a rare event in our MD sampling, corroborating the idea that the F5TPB counterions can effectively work as fine-tuning spacers able to limit tight RB-RB aggregation that induces fluorescence quenching while keeping the RB dyes comprised in large aggregates and close enough to facilitate ET processes. 

In conclusion, the presented FF for RB dyes represents a significant improvement towards the simulation of RB aggregation processes in a solution, providing atomistic details of the possible conformations of RB/counterions in solvated aggregates that could be exploited to understand how the structural details (i.e., RB spacing, reciprocal orientation, etc.) could affect the optical properties and the ET efficiency within realistic networks of RB dyes.

## Data Availability

All raw data from MD simulations (i.e., trajectories) not explicitly reported in the Supplementary Materials are freely available upon request to the authors.

## Supplementary Materials

The following supporting information can be downloaded at: https://www.mdpi.com/article/10.3390/molecules28124742/s1, Figure S1: New atom types defined for the FF parameterization of RB ethyl ester; Table S1: Cartesian coordinates, atom types, and the ESP charges for RB ethyl ester; Table S2: Cartesian coordinates, atom types and the ESP charges for F5TPB; Table S3: Equilibrium bond lengths for RB ethyl ester. Table S4: Torsional parameters for RB ethyl ester; Table S5: LJ parameters for two interacting RB molecules; Table S6: FF parameters for the F5TPB counterion; Figure S2: Continuation of simulations; Figure S3: RB-RB radial pair distribution functions; Figure S4: RB-F5TPB radial pair distribution functions.
