# Supplementary material for: Characterizing Counterion-Dependent Aggregation of Rhodamine B by Classical Molecular Dynamics Simulations
Source: Molecules. 2023 Jun 13;28(12):4742. doi: 10.3390/molecules28124742 (PMC10304572; doi:10.3390/molecules28124742)
Supplement: Supplementary file 1 [file molecules-28-04742-s001.zip › molecules-2375590-supplementary.pdf]

SUPPORTING INFORMATION

Characterizing Counterion-Dependent Aggregation of Rhodamine B by Classical Molecular Dynamics simulations

Giacomo Fanciullo [1], Silvia Orlandi [1], Andrey S. Klymchenko [2], Luca Muccioli [1,*] and Ivan Rivalta [1,3,*]

[1] Dipartimento di Chimica Industriale "Toso Montanari", Alma Mater Studiorum, Università di Bologna, Viale del Risorgimento 4, 40136 Bologna, Italy.
[2] Laboratoire de Biophotonique et Pharmacologie, UMR 7213 CNRS, Université de Strasbourg, Faculté de Pharmacie, 74, Route du Rhin, 67401 Illkirch Cedex, France
[3] ENSL, CNRS, Laboratoire de Chimie UMR 5182, 46 Allée d'Italie, 69364 Lyon France
* Correspondence: luca.muccioli@unibo.it (L.M.); i.rivalta@unibo.it (I.R.).

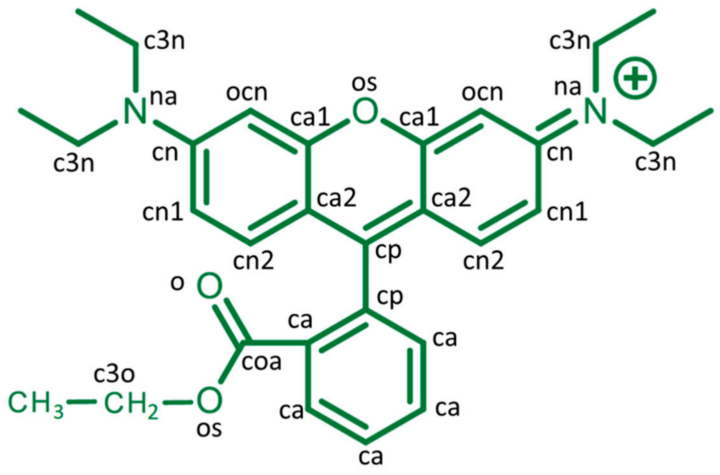

Figure S1. New atom types defined for the FF parameterization of RB ethyl ester. In this picture are reported also some of the GAFF atom types (ca, cp, o, oc, na) used for parametrizing the bond lengths. Atoms that are not labelled have been also associated to GAFF atom types (ha, hc, c3).

Table S1. Cartesian coordinates for the DFT optimized structure of RB ethyl ester along with the atom types and the (symmetrized) ESP charges (last column). All DFT calculations have been performed at the B3LYP-D3//6-311+G* level.

C1	3.607510	0.384707	-0.518499	cn1	-0.3256
C2	2.449359	1.104430	-0.623423	cn2	-0.1974
C3	1.169630	0.488647	-0.528669	ca2	0.0502
C4	1.168176	-0.919023	-0.323669	ca1	0.3885
C5	2.322891	-1.667530	-0.215941	ocn	0.5143
C6	3.592691	-1.041814	-0.316708	cn	0.4809
C7	-0.057138	1.176376	-0.600254	cp	-0.1615
C8	-1.202397	-0.961826	-0.308768	ca1	0.3885
C9	-1.256526	0.446969	-0.505981	ca2	0.0503
C10	-2.558786	1.020942	-0.556777	cn2	-0.1975
H11	-2.649567	2.093761	-0.685591	ha	0.1692
C12	-3.688942	0.260172	-0.440080	cn1	-0.3255
C13	-3.620894	-1.168710	-0.265373	cn	0.4809
C14	-2.328367	-1.750661	-0.189522	ocn	0.5144
H15	4.546174	0.916150	-0.585959	ha	0.2135
H16	2.502466	2.176463	-0.778124	ha	0.1693
H17	2.211451	-2.729278	-0.051712	ha	0.1788
H18	-4.646428	0.760394	-0.472622	ha	0.2135
H19	-2.177694	-2.808855	-0.033967	ha	0.1789
N20	4.744413	-1.761062	-0.231244	na	-0.4429
O21	-0.004852	-1.603801	-0.219177	os	-0.2513
N22	-4.744891	-1.929275	-0.176236	na	-0.4430
C23	-0.085701	2.643197	-0.869354	cp	0.3687
C24	-0.323491	3.057116	-2.185235	ca	-0.2879
C25	0.142189	4.972591	-0.216691	ca	-0.1083
C26	-0.328579	4.412905	-2.515726	ca	-0.0531
H27	-0.497701	2.308855	-2.952271	ha	0.1515
C28	-0.093882	5.372477	-1.529950	ca	-0.1604
H29	0.316976	5.712032	0.555819	ha	0.1524
H30	-0.514888	4.714679	-3.541938	ha	0.1481
H31	-0.098069	6.428605	-1.780875	ha	0.1579
C32	0.356938	3.186641	1.543864	coa	0.7492
O33	0.164216	2.053824	1.954496	o	-0.5105
O34	0.782757	4.191949	2.323369	os	-0.4519
C35	0.146337	3.613617	0.128436	ca	-0.2287
C36	-6.075130	-1.304185	-0.112911	c3n	0.3131
H37	-6.121980	-0.499538	-0.849760	hc	0.0142
H38	-6.807995	-2.039117	-0.441496	hc	0.0142
C39	-6.428413	-0.791528	1.286542	c3	-0.4092
H40	-6.429839	-1.612033	2.011750	hc	0.1184
H41	-5.707471	-0.040272	1.625172	hc	0.1184
H42	-7.424543	-0.336382	1.278826	hc	0.1184
C43	-4.599323	-3.373638	0.116292	c3n	0.3132
H44	-3.938467	-3.802751	-0.644985	hc	0.0142
H45	-4.092406	-3.485803	1.084348	hc	0.0142
C46	-5.893891	-4.180528	0.132218	c3	-0.4092
H47	-6.406300	-4.156487	-0.834460	hc	0.1184
H48	-5.632282	-5.222230	0.343307	hc	0.1184
H49	-6.587107	-3.849656	0.911137	hc	0.1184
C50	6.052050	-1.088136	-0.196346	c3n	0.3132
H51	6.807066	-1.803700	-0.517386	hc	0.0142
H52	6.062623	-0.298589	-0.950840	hc	0.0142
C53	6.402360	-0.532667	1.187495	c3	-0.4092
H54	5.658899	0.199740	1.518630	hc	0.1184
H55	6.441202	-1.336622	1.930009	hc	0.1184
H56	7.381436	-0.042688	1.158181	hc	0.1184
C57	4.653192	-3.204208	0.087406	c3n	0.3132
H58	4.158045	-3.317366	1.061464	hc	0.0142
H59	4.002322	-3.670538	-0.660614	hc	0.0142
C60	5.975989	-3.963853	0.107024	c3	-0.4092
H61	6.663069	-3.593682	0.873647	hc	0.1183
H62	5.753715	-5.010103	0.339626	hc	0.1183
H63	6.479603	-3.939763	-0.864261	hc	0.1183
C64	0.991215	3.878228	3.726331	c3o	0.3690
H65	0.049102	3.509855	4.143962	hc	0.0033
H66	1.730754	3.074579	3.795231	hc	0.0033
C67	1.460865	5.150651	4.401989	c3	-0.3096
H68	1.631684	4.959155	5.466310	hc	0.0935
H69	2.397374	5.503344	3.958451	hc	0.0935
H70	0.709202	5.940849	4.308062	hc	0.0936

Table S2. Cartesian coordinates for the DFT optimized structure of F5TPB counterion along with the atom types and the (symmetrized) ESP charges (last column). All DFT calculations have been performed at the B3LYP-D3//6-311+G* level.

B1	-0.00300	-0.00700	0.00700	b	6.3646
C2	-1.04200	1.09400	0.68000	ca	-2.1699
C3	-2.01200	1.68500	-0.13700	ca	0.4701
C4	-1.07800	1.53600	2.00300	ca	0.4701
C5	-2.93200	2.62600	0.29100	ca	0.2285
C6	-1.98600	2.47700	2.47700	ca	0.2285
C7	-2.91600	3.03400	1.61600	ca	0.1911
C8	1.09800	-0.61700	1.08300	ca	-2.1699
C9	2.02000	0.26300	1.66000	ca	0.4701
C10	1.31900	-1.95500	1.41000	ca	0.4701
C11	3.03400	-0.11600	2.52400	ca	0.2285
C12	2.32400	-2.38100	2.27300	ca	0.2285
C13	3.18600	-1.45800	2.83900	ca	0.1911
C14	-1.04600	-1.17100	-0.54500	ca	-2.1700
C15	-1.29900	-1.52200	-1.87100	ca	0.4702
C16	-1.86900	-1.82300	0.37800	ca	0.4702
C17	-2.24900	-2.46500	-2.25000	ca	0.2286
C18	-2.82500	-2.76800	0.04600	ca	0.2286
C19	-3.01800	-3.09600	-1.28800	ca	0.1911
C20	0.94500	0.63100	-1.18400	ca	-2.1700
C21	1.06600	1.97500	-1.53500	ca	0.4702
C22	1.81600	-0.21600	-1.87600	ca	0.4702
C23	1.94100	2.44000	-2.51100	ca	0.2286
C24	2.70100	0.20200	-2.85500	ca	0.2286
C25	2.76400	1.55000	-3.18000	ca	0.1912
F26	-3.83000	3.97000	2.07600	f	-0.2293
F27	-3.85600	3.17600	-0.58600	f	-0.2293
F28	-1.97400	2.86200	3.81000	f	-0.2293
F29	-0.22800	1.00300	2.97100	f	-0.2859
F30	-2.05900	1.35100	-1.49300	f	-0.2859
F31	-1.72900	-1.53600	1.73800	f	-0.2859
F32	0.54600	-2.97800	0.86300	f	-0.2859
F33	1.93300	1.62700	1.37200	f	-0.2859
F34	3.89900	0.82100	3.07100	f	-0.2293
F35	4.19400	-1.86900	3.69800	f	-0.2293
F36	2.48300	-3.72900	2.56200	f	-0.2293
F37	-3.59400	-3.38500	1.02400	f	-0.2293
F38	-3.97000	-4.03600	-1.65000	f	-0.2293
F39	-2.44900	-2.76900	-3.59000	f	-0.2293
F40	0.31200	2.96100	-0.90000	f	-0.2859
F41	2.00900	3.79200	-2.81400	f	-0.2293
F42	3.64400	1.99800	-4.15300	f	-0.2293
F43	3.52500	-0.70100	-3.51100	f	-0.2294
F44	1.80400	-1.58400	-1.59600	f	-0.2858
F45	-0.61700	-0.91600	-2.92500	f	-0.2858

Table S3. Equilibrium bond lengths that have been re-parametrized for the RB ethyl ester. The corresponding GAFF force constants are also reported for completeness.

Pair of atom types	Force constant (kcal mol^{-1} $Å^{-2}$)	Equilibrium length (Å)
coa o	648.0	1.2407
coa ca	349.7	1.4870
coa os	411.3	1.3650
c3o os	301.5	1.4756
c3o hc	337.3	1.0920
c3o c3	303.1	1.5350
ca2 cp	466.1	1.4094
ca2 ca1	478.4	1.4228
ca1 os	372.4	1.3865
cn2 ca2	478.4	1.4252
cn2 ha	344.3	1.0870
cn2 cn1	478.4	1.3688
cn1 ha	344.3	1.0870
cn cn1	478.4	1.4420
cn na	470.3	1.3500
ocn cn	478.4	1.4210
ocn ha	344.3	1.0870
ocn ca1	478.4	1.3870
c3n na	334.7	1.4860
c3n c3	303.1	1.5350
c3n hc	337.3	1.0920

Table S4. New parameters describing the three torsions whose potentials have been re-parametrized for the RB ethyl ester. The n values indicate the different periodicity contributions, while the V_n values are the corresponding amplitudes (kcal/mol), according to the CHARMM potential energy function.

Xanthene-ammine torsion (X – cn – na – X)

n	2	4	8	10	12
V_n	-1.7024	0.0224	-0.1055	0.0543	-0.0672

Xanthene-phenyl torsion (X – cp – cp – X)

n	2	4	6	8
V_n	-1.6414	0.1914	-0.0582	-0.1644

Phenyl-carboxyl torsion (X – ca – coa – X)

n	2	4	6	8	10
V_n	-1.591	0.0192	-0.0201	-0.0326	-0.0314

Table S5. New LJ parameters describing the interactions between couple of atoms in two interacting RB molecules. These values correctly reproduce the energy of two RBs approaching to form a stacked H dimer as predicted by DFT (B3LYP-D3//6-311+G* level of theory). The associated GAFF $2r_{min}$ values are also reported for completeness.

Pair of atom types	ε (kcal/mol)	$2r_{min}$ (Å)
os na	0.34000	3.50770
os ca1	0.24182	3.59170
os ca2	0.24182	3.59170
os ocn	0.24182	3.59170
os cn	0.24182	3.59170
os cn1	0.24182	3.59170
os cn2	0.24182	3.59170
na ca1	0.24182	3.73200
na ca2	0.24182	3.73200
na ocn	0.24182	3.73200
na cn	0.24182	3.73200
na cn1	0.24182	3.73200
na cn2	0.24182	3.73200
ca1 ca2	0.17198	3.81600
ca1 ocn	0.17198	3.81600
ca1 cn	0.17198	3.81600
ca1 cn1	0.17198	3.81600
ca1 cn2	0.17198	3.81600
ca2 ocn	0.17198	3.81600
ca2 cn	0.17198	3.81600
ca2 cn1	0.17198	3.81600
ca2 cn2	0.17198	3.81600
ocn cn	0.17198	3.81600
ocn cn1	0.17198	3.81600
ocn cn2	0.17198	3.81600
cn cn1	0.17198	3.81600
cn cn2	0.17198	3.81600
cn1 cn2	0.17198	3.81600

Table S6. FF parameters introduced for the F5TPB counterion. The B-C bond equilibrium length and force constant have been determined from DFT calculations (B3LYP-D3//6-311+G* level of theory). For bending and torsion angles involving boron, the corresponding parameters for tetra-phenyl methane (taken from ref. [24]) have been considered. The LJ parameters for boron are also taken from ref. [24].

B-C bond

Pair of atom types	Force constant (kcal mol^{-1} Å^{-2})	Equilibrium length (Å)
b ca	363.8	1.3440

Bending angles involving boron

Triplet of atom types	Force constant (kcal mol^{-1} rad^{-2})	Equilibrium angle (deg)
b ca ca	63.840	120.630
ca b ca	217.48	100.140

Torsion angles involving boron (X – b – ca – X)

n	6
V_n	0

LJ parameters for boron

ε (kcal/mol)	$2r_{min}$ (Å)
0.14	4.30

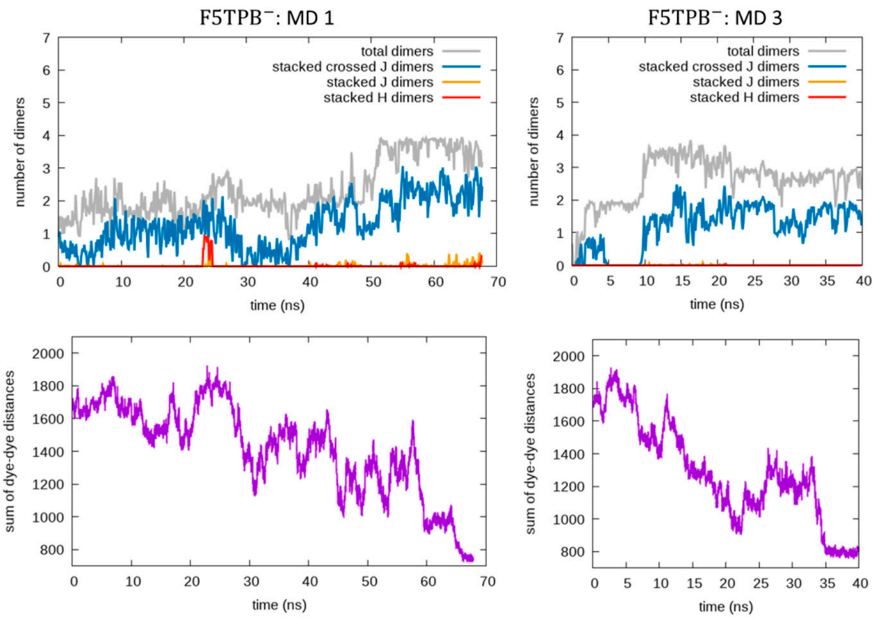

Figure S2. Prolongation of the simulations MD 1 and MD 3 reported in Figure 5 of the main text.

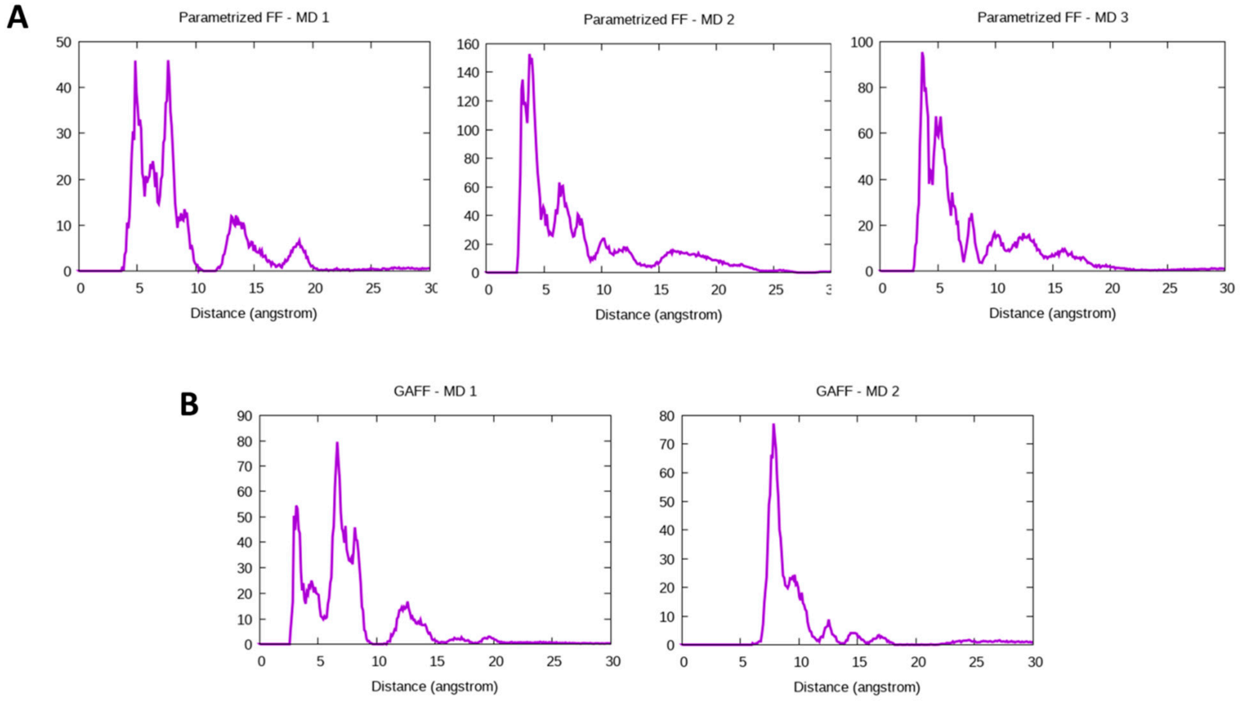

Figure S3. RB-RB radial pair distribution functions obtained from the last nanosecond of the simulations (with F5TPB counterion): comparison between the re-parametrized FF (panel A) and GAFF (panel B).

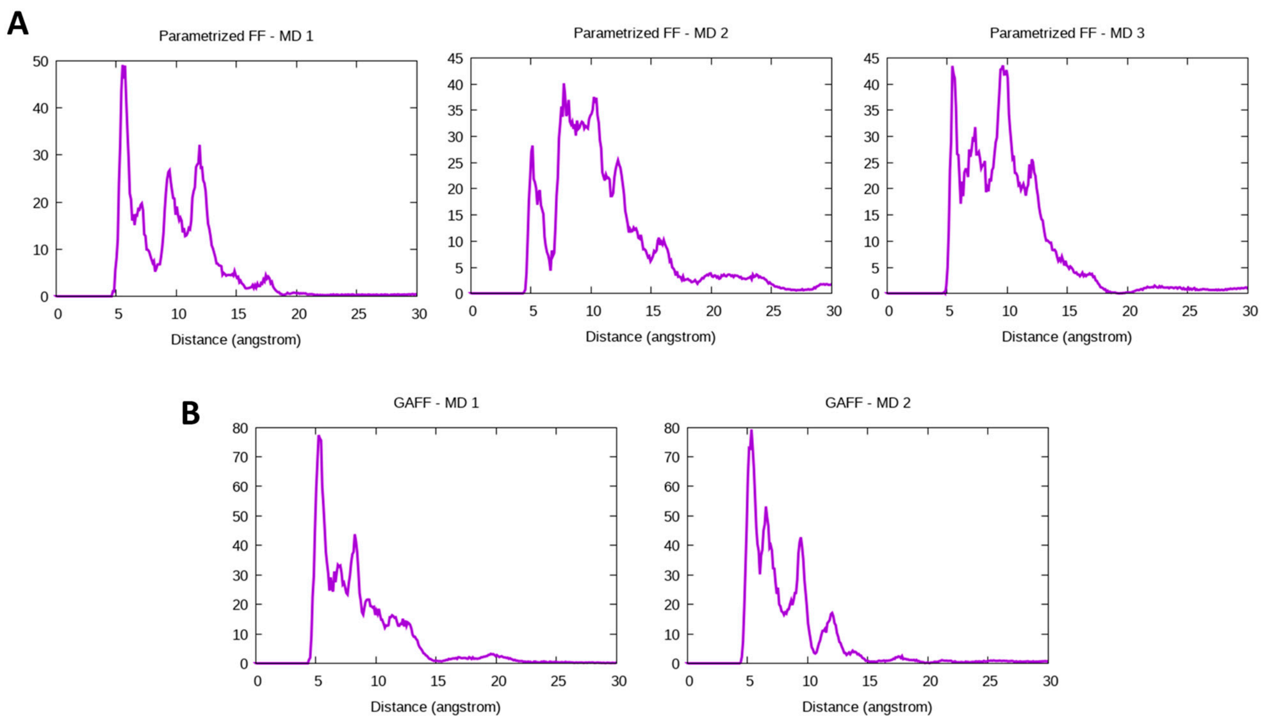

Figure S4. RB-F5TPB radial pair distribution functions obtained from the last nanosecond of the simulations: comparison between the re-parametrized FF (panel A) and GAFF (panel B).
